# Predicting recurrence of *Clostridium difficile* infection following encapsulated fecal microbiota transplantation

**DOI:** 10.1186/s40168-018-0549-6

**Published:** 2018-09-18

**Authors:** Christopher Staley, Thomas Kaiser, Byron P. Vaughn, Carolyn T. Graiziger, Matthew J. Hamilton, Tauseef ur Rehman, Kevin Song, Alexander Khoruts, Michael J. Sadowsky

**Affiliations:** 1 Department of Surgery, University of Minnesota, Minneapolis, MN USA; 2 BioTechnology Institute, University of Minnesota, 140 Gortner Lab, 1479 Gortner Ave, St. Paul, MN 55108 USA; 3 Division of Gastroenterology, Department of Medicine, University of Minnesota, Minneapolis, Minnesota USA; 4 Department of Soil, Water and Climate, University of Minnesota, St. Paul, Minnesota USA; 5 Department of Plant and Microbial Biology, University of Minnesota, St. Paul, Minnesota USA

**Keywords:** *Clostridium difficile*, Encapsulated microbiota, Fecal microbiota transplantation, Machine learning, Prediction model, Microbial community structure

## Abstract

**Background:**

Fecal microbiota transplantation (FMT) is an effective treatment for recurrent *Clostridium difficile* infection (rCDI). The use of freeze-dried, encapsulated donor material for FMT (cap-FMT) allows for an easy route of administration and remains clinically effective in the majority of rCDI patients. We hypothesized that specific shifts in the microbiota in response to cap-FMT could predict clinical outcome. We further evaluated the degree of donor microbiota engraftment to determine the extent that donor transfer contributed to recovery.

**Results:**

In total, 89 patients were treated with 100 separate cap-FMTs, with a success rate (no rCDI 60 days post cap-FMT) of 80%. Among responders, the lower alpha diversity (ANOVA *P* < 0.05) observed among patient’s pre-FMT samples was restored following cap-FMT. At 1 week post-FMT, community composition varied by clinical outcome (ANOSIM *P* < 0.001), with similar abundances among families (*Lachnospiraceae*, *Ruminococcaceae*, and *Bacteroidaceae*) in responder and donor samples. Families that showed differential abundances by outcome (response vs. recurrence) from samples collected 7 days following cap-FMT were used to construct a regression tree-based model to predict recurrence. Results showed a training accuracy of 100% to predict recurrence and the model was 97% accurate against a test data set of samples collected 8–20 days following cap-FMT. Evaluation of the extent of engraftment using the Bayesian algorithm SourceTracker revealed that approximately 50% of the post-FMT communities of responders were attributable to donor microbiota, while an additional 20–30% of the communities were similar to a composite healthy microbiota consisting of all donor samples.

**Conclusions:**

Regression tree-based analyses of microbial communities identified taxa significantly related to clinical response after 7 days, which can be targeted to improve microbial therapeutics. Furthermore, reinstatement of a healthy assemblage following cap-FMT was only partially attributable to explicit donor engraftment and continued to develop towards an overall healthy assemblage, independent of donor.

**Electronic supplementary material:**

The online version of this article (10.1186/s40168-018-0549-6) contains supplementary material, which is available to authorized users.

## Background

*Clostridium difficile* infection (CDI) remains a common cause of hospital and community-acquired infection [1]. One of the most difficult clinical challenges associated with CDI is recurrent infection (rCDI) despite multiple courses of antibiotics [2, 3]. Fecal microbiota transplantation (FMT) has emerged recently as a highly effective treatment of rCDI that is now endorsed by professional societies and incorporated into standard treatment guidelines as an option following failure of antibiotic treatment [4, 5]. FMT involves transfer of fecal microorganisms from healthy donors to patients to correct antibiotic-induced dysbiosis, which is the primary causal risk factor for CDI in most patients.

Unlike antibiotics, FMT represents a restorative therapeutic approach that results in donor-like normalization of fecal microbial community structure and functionality [6, 7]. However, despite its impressive overall efficacy in breaking the cycle of CDI recurrence, FMT fails to cure rCDI in a fraction of patients. The reasons for FMT failure are not well understood; potential variables include specific patient factors [8, 9], potential resistance of individual strains of *C. difficile* bacteria to FMT activity [9], or failure to activate protective mechanisms or achieve full potency, possibly due to inadequate engraftment of key donor microorganisms [10]. In order to understand why FMT may fail, it is critical to know the mechanisms by which FMT is able to break the cycle of CDI recurrence. Achieving this understanding is also important for development of reliable next-generation anti-CDI therapeutics.

Identification of specific microbial taxa that are essential for resolution of rCDI would allow for a targeted microbiota restoration approach and decrease the FMT failure rate. Specific approaches have included attempts to correlate microbiome analyses with CDI recurrence risk, as well as treatments of rCDI with defined consortia of microorganisms or preparations of fecal microbiota of reduced complexity [11–14]. However, these investigations have not yielded consistent results. Here, we expand upon our previous studies analyzing microbiome recovery in rCDI patients treated with an encapsulated preparation of freeze-dried microbiota (cap-FMT) [15]. Clinically, cap-FMT has been very successful [15, 16]. However, in contrast to colonoscopic administration of fecal microbiota, which results in prompt engraftment of the entire donor bacterial communities within 24–48 h [17], treatment with this oral FMT preparation is associated with more gradual, punctuated kinetics of microbiome normalization over a period of approximately a month [18]. The increased period of recovery presents a potential opportunity to capture the essential steps in microbiome repair and identify critical microbial taxa in controlling CDI.

## Results

### Clinical cohort and fecal samples

The current analysis includes samples from an expanded cohort of 89 consecutively treated rCDI patients who participated in the study. The cohort includes the 49 patients described previously [15] and reflects 100 separate cap-FMTs from eight different donor lots. A donor lot is defined as the preserved fecal microbiota purified from a single stool sample. The demographics and clinical characteristics of this patient cohort are shown in Additional file 1: Table S1. None of the patients had underlying inflammatory bowel disease (IBD), which is associated with lower success rates of FMT in treating rCDI [19]. The success rate for cap-FMT treatment was 80%. Among the non-responders, the median interval between cap-FMT and diagnosis of CDI relapse was 13 days (range 4–42 days). The general clinical course among patients following cap-FMT was similar to that following colonoscopic FMT experience in our center (> 400 colonoscopic FMTs for rCDI), including the interval between the procedure and diagnosis of CDI relapse in patients without IBD who failed treatment. Patients who had CDI recurrence were subsequently retreated by cap-FMT, received colonoscopic FMT, or remained on a long-term suppressive treatment with a daily dose of vancomycin. During the course of the study, the donor lot, total capsule dosage, and timing of delivery varied based on clinical experience, but none of these factors were shown to be significantly related to patient outcomes (Additional file 1: Table S2). Due to variation in study protocols during the course of this study and logistics of stool collections, analyses were done using samples that were grouped by broad time points encompassing days (days 2–6), weeks (days 7–20), months (days 21–60), and longer-term samples (> 60 days) following cap-FMT (Additional file 1: Figure S1).

### FMT is associated with characteristic shifts in bacterial community alpha and beta diversity

**Fig. 1:**
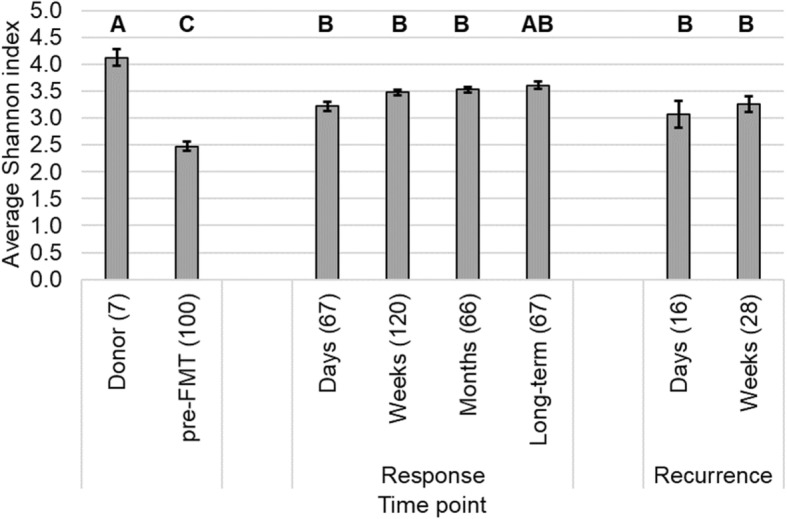
Shannon indices of donor and patient communities. The number of samples (*n*) is shown in parentheses. Letters indicate significant differences as assessed by Tukey’s *post hoc* test (*P* < 0.05), where sample groups sharing the same letter did not differ significantly

**Fig. 2:**
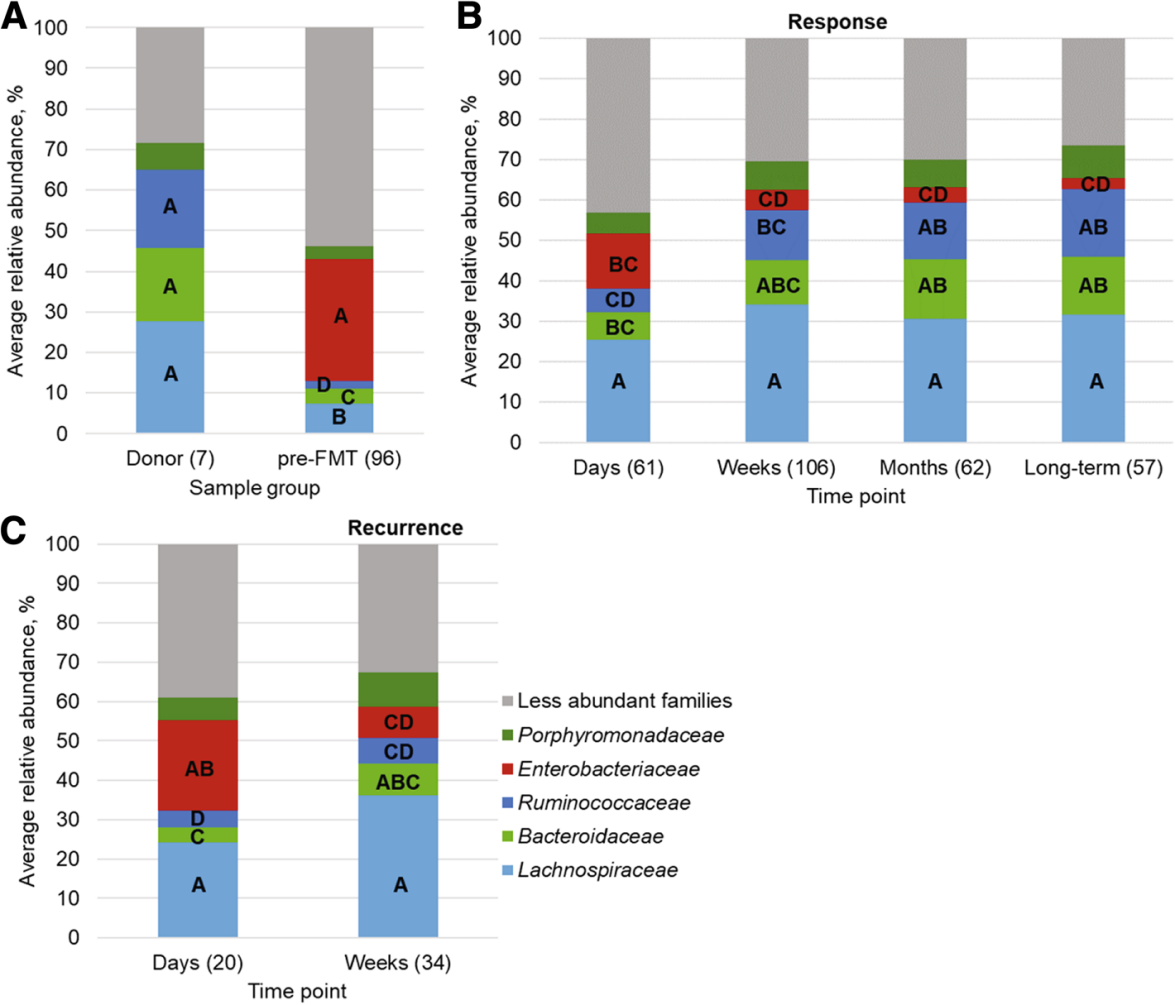
Distribution of abundant phyla among **A**) donor and pre-FMT samples, **B**) post-FMT samples from responders, and **C**) post-FMT samples from patients who had recurrence. Numbers in parentheses reflect the numbers of samples in each group. Letters on family abundances reflect significant differences by Tukey’s *post hoc* test (*P* < 0.05) across all groups (all panels) for each family, separately. No significant differences were observed for abundances of *Porphyromonadaceae*. Less abundant families represent those present at mean abundance ≤ 5% of sequence reads among all samples

As expected, alpha diversity, community richness, and evenness measured by the Shannon index were lowest in pre-FMT samples compared to those collected from donors and patients post-FMT (*post hoc P* ≤ 0.014; Fig. 1). In addition, post-FMT samples from non-responders had lower alpha diversity relative to samples from donors (*P* ≤ 0.034), but did not differ significantly from responder post-FMT samples, at any time point. Donor communities were primarily comprised of members of the *Lachnospiraceae*, *Ruminococcaceae*, *Bacteroidaceae*, and *Porphyromonadaceae*, while pre-FMT communities had greater numbers of *Enterobacteriaceae* (Fig. 2). The microbial communities in responders began to taxonomically resemble those of the donors at the “weeks” time point post-FMT (Fig. 2), with increases in the donor-associated families described above, and a significant reduction in abundances of members of the *Enterobacteriaceae*.

Changes in bacterial community composition were evaluated by using Bray-Curtis dissimilarities, which account for differences in the abundances of operational taxonomic units (OTUs) among sample groups. Over time, progressive, temporal changes were observed in bacterial communities in post-FMT samples from patients who responded to cap-FMT, but not those who experienced recurrence. No significant differences were noted between the fecal bacterial communities in cap-FMT responders and non-responders at the “days” time point (analysis of similarity (ANOSIM) *R* = 0.10, *P* = 0.052). However, the communities from cap-FMT responders and non-responders diverged after the “weeks” time points (*R* = 0.11, *P* = 0.020; Bonferroni-corrected *α* = 0.002). Specifically, bacterial communities of cap-FMT responders differed significantly at all later time points (“weeks” through “long-term”) relative to the communities at the “days” time point (*R* = 0.14–0.23, *P* < 0.001). Moreover, fecal bacterial composition of responder samples did not change significantly following the “weeks” time point (*R* = − 0.03 to − 0.01, *P* ≥ 0.627). In contrast, no significant differences between the “days” and “weeks” time points (*R* = 0.05, *P* = 0.142) were noted in the bacterial communities in feces from the cap-FMT non-responder group.

To evaluate which metabolic functional traits may be associated with response to cFMT, functional inferences were performed and correlated with days-post-FMT among samples from patients who responded. The abundances of most inferred functional traits were negatively correlated with the number of days following cFMT (Additional file 1: Table S3), including genes associated with nitrogen and sulfur metabolism (*ρ* = − 0.124 and − 0.158, *P* = 0.038 and 0.008, respectively). However, functional genes within the tier 2 category of glycan biosynthesis and metabolism showed weak, but significant positive correlations (*ρ* = 0.125–0.191, *P* ≤ 0.037). Notably, genes inferred to function in primary and secondary bile acid biosynthesis were also positively correlated with duration following cFMT among responders (*ρ* = 0.215 and 0.217, respectively; *P* < 0.0001). Among genes involved in secondary bile acid, choloylglycine hydrolase (K01442), 7α-hydroxysteroid dehydrogenase (*hdhA*; KO00076), and 3-dehydro-bile acid Δ4,6-reductase (*baiN*; KO07007) were inferred. Members of the *Lachnospiraceae* were the predominant contributors of these genes among all samples (56.1, 93.9, and 66.5%, respectively). Members of the *Bacteroidaceae*, *Porphyromonadaceae*, *Verrucomicrobiaceae*, and *Ruminococcaceae* were also predominant contributors to abundances of choloylglycine hydrolase (9.1, 7.6, 6.9, and 6.6%, respectively), and members of *Enterobacteriaceae* and *Ruminococcaceae* were predominant contributors to abundances of *baiN* (10.5 and 8.1%). Members of *Coriobacteriaceae* and *Synergistaceae* were among the only other families inferred to contribute to abundances of *hdhA* (4.3 and 1.1%).

### Identification of markers indicative of clinical outcome

**Fig. 3:**
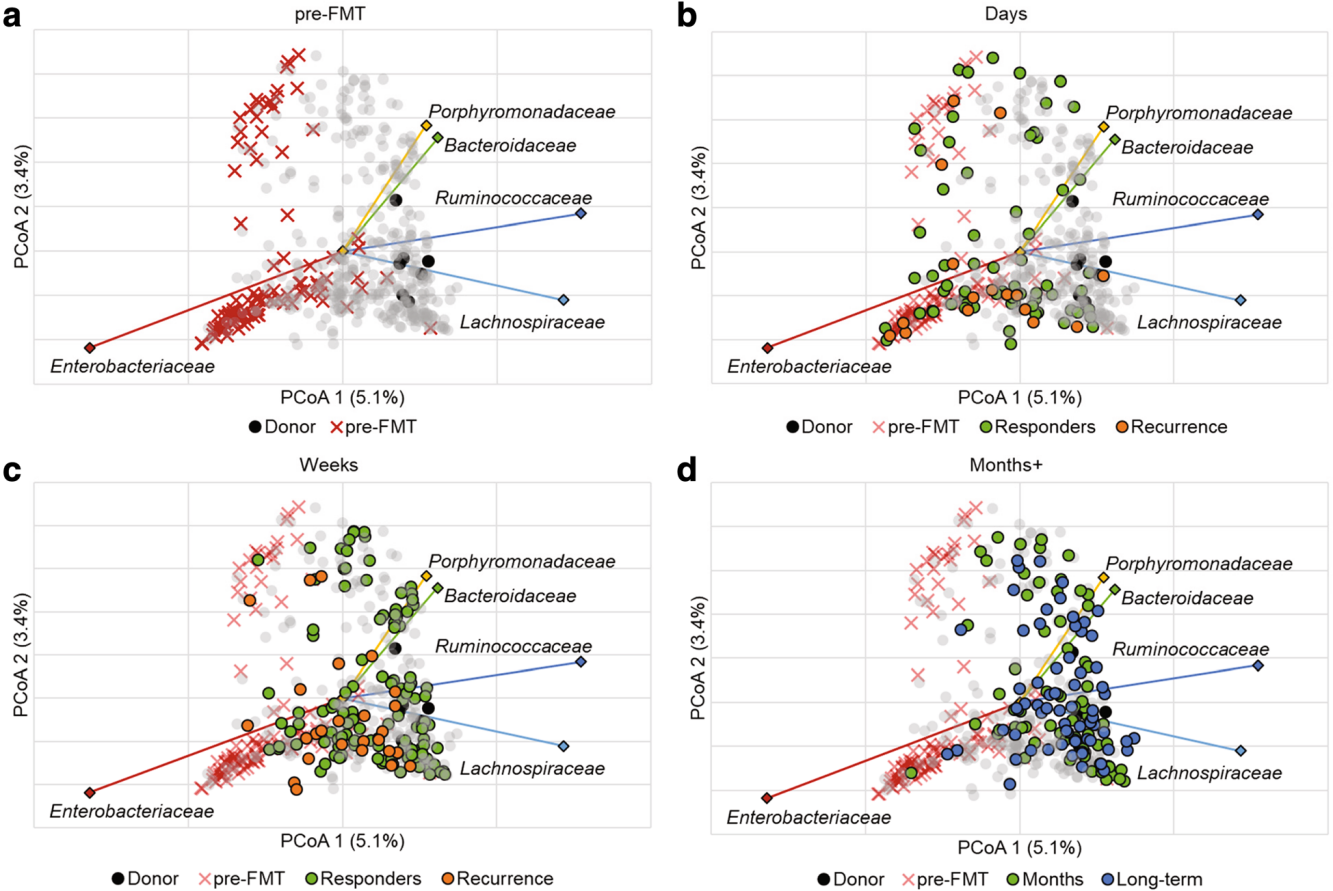
Ordination of Bray-Curtis dissimilarity matrices by PCoA (*r*^2^ = 0.23) and the five most abundant families. Panels reflect the same analysis but are colored by time point for clarity: **a**) pre-FMT, **b**) days (2–6 days post-FMT), **c**) weeks (7–20 days), and **d**) months (21–60 days) and long-term (> 60 days). A total of 471 axes were used to explain all variation with the remaining axes explain < 2.3% of the variation individually. Family abundances were significantly related to *x*-axis position by Spearman correlation (*P* < 0.05). Legend: black circle—donor, ex mark—pre-FMT, green circle—responder/months, orange circle—recurrence, blue circle—long-term, gray circle—sample not associated with time point

Ordination of Bray-Curtis dissimilarity matrices among samples by principal coordinate analysis (PCoA, Fig. 3) showed separation of samples associated with time and clinical outcome along the *x*-axis. This is in agreement with ANOSIM analyses (above). The *Lachnospiraceae*, *Ruminococcaceae*, *Bacteroidaceae*, *Porphyromonadaceae*, and *Enterobacteriaceae* comprised the predominant families whose abundances were significantly correlated with position along this axis (Fig. 3). Similarly, and more specifically, the predominant OTUs that showed significantly different abundances due to time point and were related to clinical outcome by linear discriminant analysis of effect size (LEfSe), were also primarily classified to genera within the *Lachnospiraceae*, *Ruminococcaceae*, and *Bacteroidaceae*, among others (Table 1).

**Table 1:** Genus-level classification and relative abundances of OTUs found to be significantly indicative (LDA ≥ 2.0, *P* < 0.05) of time point and clinical outcome by LEfSe analysis. Only the 15 most abundant genera are shown

Family	Genus			Response				Recurrence	
		Donor	Pre-FMT	Days	Weeks	Months	Long-term	Days	Weeks
*Bacteroidaceae*	*Bacteroides*	13.1 ± 3.3	2.1 ± 0.4	0.5 ± 0.1	0.1 ± 0.1	2.7 ± 0.3	1.9 ± 0.4	2.7 ± 0.8	1.5 ± 0.5
	*Parabacteroides*	3.2 ± 1.0	0.9 ± 0.5	0.0 ± 0.0	8.5 ± 0.9	0.2 ± 0.1	0.2 ± 0.1	0.1 ± 0.0	0.2 ± 0.1
*Coriobacteriaceae*	*Collinsella*	5.7 ± 1.3	0.6 ± 0.4	0.5 ± 0.1	5.0 ± 0.8	1.3 ± 0.4	2.1 ± 0.5	0.9 ± 0.2	0.8 ± 0.2
*Enterobacteriaceae*	*Esherichia/Shigella*	9.4 ± 1.1	0.7 ± 0.2	1.0 ± 0.2	2.5 ± 0.5	2.1 ± 0.3	3.2 ± 0.4	1.1 ± 0.2	1.3 ± 0.1
*Lachnospiraceae*	*Anaerostipes*	13.0 ± 1.6	0.9 ± 0.4	1.3 ± 0.3	1.6 ± 0.4	2.1 ± 0.3	2.6 ± 0.4	0.7 ± 0.1	1.3 ± 0.2
	*Blautia*	12.3 ± 1.4	1.3 ± 0.4	1.1 ± 0.2	1.8 ± 0.6	2.7 ± 0.4	2.9 ± 0.4	0.7 ± 0.1	1.4 ± 0.2
	*Coprococcus*	3.4 ± 1.8	0.5 ± 0.5	0.8 ± 0.5	10.6 ± 3.4	2.0 ± 1.1	0.9 ± 0.5	0.5 ± 0.2	0.7 ± 0.4
	*Dorea*	7.9 ± 2.1	0.0 ± 0.0	1.1 ± 0.3	5.3 ± 1.5	1.8 ± 0.5	2.1 ± 0.8	0.7 ± 0.2	1.0 ± 0.3
	*Fusicatenibacter*	13.1 ± 3.3	2.1 ± 0.4	0.5 ± 0.1	0.1 ± 0.1	2.7 ± 0.3	1.9 ± 0.4	2.7 ± 0.8	1.5 ± 0.5
	*Moryella*	3.2 ± 1.0	0.9 ± 0.5	0.0 ± 0.0	8.5 ± 0.9	0.2 ± 0.1	0.2 ± 0.1	0.1 ± 0.0	0.2 ± 0.1
	*Roseburia*	5.7 ± 1.3	0.6 ± 0.4	0.5 ± 0.1	5.0 ± 0.8	1.3 ± 0.4	2.1 ± 0.5	0.9 ± 0.2	0.8 ± 0.2
*Oscillospiraceae*	*Oscillibacter*	9.4 ± 1.1	0.7 ± 0.2	1.0 ± 0.2	2.5 ± 0.5	2.1 ± 0.3	3.2 ± 0.4	1.1 ± 0.2	1.3 ± 0.1
*Rikenellaceae*	*Alistipes*	13.0 ± 1.6	0.9 ± 0.4	1.3 ± 0.3	1.6 ± 0.4	2.1 ± 0.3	2.6 ± 0.4	0.7 ± 0.1	1.3 ± 0.2
*Ruminococcaceae*	*Faecalibacterium*	12.3 ± 1.4	1.3 ± 0.4	1.1 ± 0.2	1.8 ± 0.6	2.7 ± 0.4	2.9 ± 0.4	0.7 ± 0.1	1.4 ± 0.2
*Veillonellaceae*	*Veillonella*	3.4 ± 1.8	0.5 ± 0.5	0.8 ± 0.5	10.6 ± 3.4	2.0 ± 1.1	0.9 ± 0.5	0.5 ± 0.2	0.7 ± 0.4

Since members of the *Lachnospiraceae, Ruminococcaceae*, *Bacteroidaceae*, *Porphyromonadaceae*, and *Enterobacteriaceae* were related to time points and clinical outcome by various statistical tests, we hypothesized that abundances of these families could be used to predict the likelihood of recurrence. Given a statistical similarity of the microbial community composition in responders at the “weeks” time point, relative to donors, these data were selected to develop a chi-squared automatic interaction detection (CHAID)-based regression tree model, using a machine learning approach. The model was trained on all cap-FMT patient data (responders and non-responders) collected from day 7 (*n* = 64) and tested on subsequent patient data from days 8–20 post-FMT (*n* = 67, Table 2). Ten samples from the training data were withheld for validation prior to testing. Thus, the training and test datasets were independent from each other. When more than one sample representing a unique cap-FMT delivery was present in the days 8–20 dataset, only the earliest time point post-FMT was included.

**Table 2:** Confusion matrices showing the accuracy of CHAID-regression tree model to (A) training data (post-FMT day 7 composition), (B) validation data (using 10 samples withheld from the training data) and (C) test data (post-FMT day 8–20 composition)

From/to	Response	Recurrence	Total	% Correct
A				
Response	44	0	44	100
Recurrence	0	10	10	100
Total	44	10	54	100
B				
Response	6	0	6	100
Recurrence	3	1	4	25.0
Total	9	1	10	70.0
C				
Response	55	2	57	96.5
Recurrence	0	10	10	100
Total	55	12	67	97.0

The model generated had an overall training accuracy of 100% (correct classification of all samples) using the 7-day data and a test accuracy of 97.0% against the days 8–20 data set (Table 2). The model specificity was 96.5% to identify a recurrence within weeks of cap-FMT, with a sensitivity of 100% against the test data. Attempts to improve accuracy against the test data were unsuccessful by using all family-level data and by using the same parameters for tree construction, producing a maximum test accuracy of 94.0%.

### Evaluation of engraftment in relation to clinical outcome

To determine how donor microbiota engraftment influenced clinical outcome, the extent of engraftment was assessed using SourceTracker software, which employs a Bayesian algorithm to determine the percent similarity in OTU composition between the donor (the source) and patient (the sink) communities [20]. Engraftment among patient samples was assessed by (1) using communities from all donor lots as a “composite” source (evaluates the similarity of patient sample communities to a generalized healthy fecal microbiota); (2) designating each “specific” donor lot as a unique source (testing the ability of SourceTracker to discriminate among donors); and (3) analyzing “individual” donor lots and associated patient samples separately (determines the similarity to the specific donor lot representing empirical engraftment). Among all patient samples, the extent of engraftment based on these analysis methods were in the order composite > individual > specific, with each method significantly different from the others (Tukey’s *post hoc P* < 0.0001). Furthermore, since all the same OTUs were not consistently transferred to all patients, regardless of the donor lot or the method of analysis (data not shown), taxonomic assignments were performed at the family level to assess patterns of engraftment (Additional file 1: Table S4).

**Fig. 4:**
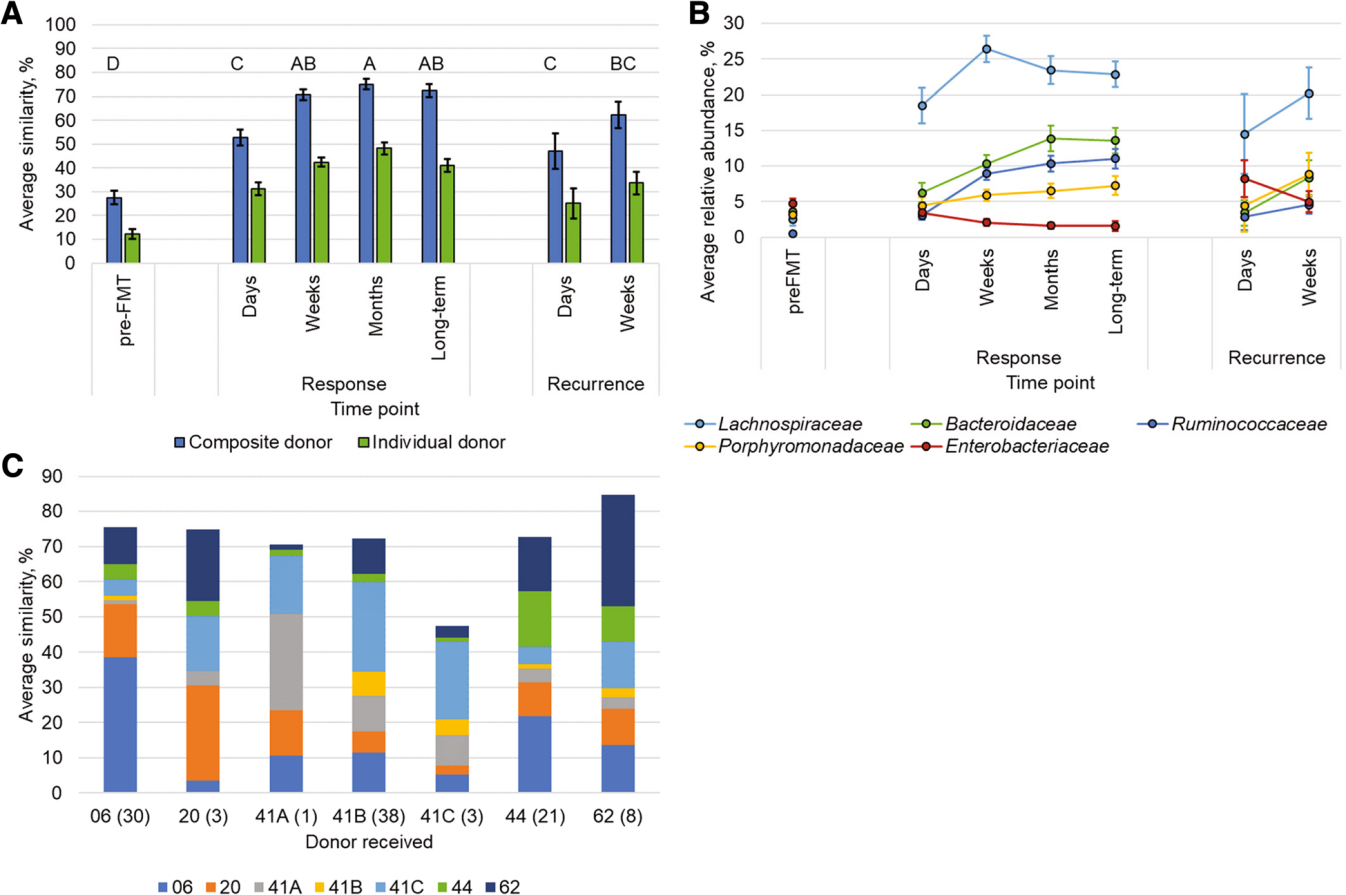
SourceTracker analysis of engraftment in patient samples. **A**) average engraftment in patient samples with analysis performed using all donors pooled (“composite”, blue) or only samples associated with a single donor lot (“individual”, green). Letters indicated significant differences by Tukey’s *post hoc* test (*P* < 0.05) for the composite analysis only, where sample groups sharing the same letter did not differ significantly. **B**) Classification and abundance of OTUs determined to engraft by SourceTracker in the composite analysis. **C**) Similarity of responder fecal communities to unique donor lots at the ‘weeks’ time point (separate analysis than that shown in **A**). Numbers in parentheses reflect the number of samples. Fecal material from donor 42 was not available for sequencing. In all panels, error bars reflect SEM

When microbial communities from all donor lots were pooled as a single composite source (Fig. 4a), approximately one quarter (27.6 ± 2.8%) of pre-FMT communities included OTUs shared with donor samples. This percentage similarity, however, was significantly less than all other post-FMT sample groups (*post hoc P* < 0.001). In contrast, among the post-FMT samples, the extent of engraftment was significantly greater at the “weeks” and later time points among patients who responded, relative to those that had recurrence within days of cap-FMT (*P* ≤ 0.036). The greatest similarity to all donors was observed among responders at approximately the 1 month post-FMT time point. Furthermore, the percent of donor engraftment was significantly and positively correlated with the number of days post-FMT (Spearman’s *ρ* = 0.536, *P* < 0.0001). The OTUs that were associated with engraftment (Fig. 4b) were predominantly classified within the families *Lachnospiraceae*, followed by *Bacteroidaceae* and *Ruminococcaceae*.

Assessment of specific donor engraftment among the entire dataset similarly revealed a small attribution (6.6 ± 1.4%) of the communities in pre-FMT samples to donor communities. This was significantly less than that seen in post-FMT engraftment samples among patients who responded to cap-FMT (*P* ≤ 0.011). Differences in engraftment between the pre-FMT samples and those from patients who had a recurrence, however, did not differ significantly (*P* ≥ 0.138). While the percent of engraftment was still significantly correlated with days post-FMT (*ρ* = 0.296, *P* < 0.0001), the strength of this relationship was much weaker compared to that obtained from analyses of the composite. Furthermore, when samples were grouped by time point, there were no significant increases in engraftment at later times (*P* ≥ 0.336). Among patient samples from responders, collected at the “weeks” time point, microbial communities showed a greater proportion of similarity to the specific donor lot the patient received (Fig. 4c, Additional file 1: Figure S2), rather than to the communities from other donor lots. However, the empirical donor lot used for cap-FMT was not assigned a significantly greater percentage of the community than lots not delivered to the patient (*post hoc P* > 0.05).

Intermediate engraftment percentages were found when an individual donor lot was compared to samples from only those patients who received that lot (Fig. 4a, Table 3). Similar to the prior analyses, this analysis indicated the percent of engraftment in pre-FMT samples was significantly less than that of all post-FMT samples (*P* ≤ 0.044). In addition, the percent of engraftment was significantly correlated with the number of days post-FMT (*ρ* = 0.473, *P* < 0.0001). Moreover, samples from patients who responded to cap-FMT also had significantly greater percentages of engraftment in the “weeks” time point, relative to those from patients who had recurrence within days of receiving cap-FMT (*P* < 0.042). While the OTUs that engrafted were classified to similar families as those observed for the composite donor (Fig. 4b), donor-specific differences in the abundance of these taxa were observed (Additional file 1: Table S3). Notably, for two donors (numbers 44 and 62), approximately 8–14% of the donor-associated community was comprised of *Verrucomicrobia*, which was not contributed from other donors, although transfer of this family did not significantly affect efficacy of these donor lots (Additional file 1: Table S2). Moreover, many taxa were not unique to a single donor lot and 23.5 to 84.6% of sequence reads in a single donor lot belonged to taxa found in one or more other donor lot (Table 4).

**Table 3:** Similarity (%) to individual donor lots. Similarity was assessed only on patients who were treated using the individual donor lot. Fecal material from donor 42 was not available for sequencing

Clinical outcome	Time point	06	20	41A	41B	41C	44	62
	n*	22	3	1	30	5	19	10
	pre-FMT	13.3 ± 4.6	18.7 ± 16.0	67.0	6.1 ± 1.8	8.2 ± 9.2	12.0 ± 4.5	25.0 ± 6.9
Response	Days	34.5 ± 5.8	45.9 ± 14.4	50.3	16.7 ± 3.9	24.2 ± 1.6	35.0 ± 5.7	51.7 ± 9.2
	Weeks	49.8 ± 4.4	47.0 ± 17.1	56.4	34.8 ± 2.7	23.9 ± 17.0	45.0 ± 5.3	52.3 ± 5.1
	Months	55.0 ± 5.0	NC	NC	39.2 ± 3.9	57.8	57.1 ± 5.1	47.5 ± 6.8
	Long-term	53.8 ± 6.2	NC	38.2	34.2 ± 3.2	73.4	42.2 ± 5.1	NC
Recurrence	Days	29.0 ± 12.7	NC	NC	23.2 ± 9.4	36.5 ± 51.6	22.6 ± 11.1	37.8 ± 12.4
	Weeks	42.0 ± 7.9	NC	NC	26.9 ± 5.8	34.8 ± 26.6	35.3 ± 6.9	53.9 ± 10.9

*NC* not collected

*n refers to total number of cap-FMTs performed using each donor lot

**Table 4:** Similarity (%) among donor lots determined by SourceTracker. Sequence data were not available for donor 42

From/to	06	20	41A	41B	41C	44	62
06	100.0	53.0	37.4	51.0	54.6	48.1	25.6
20	52.7	100.0	26.3	34.5	56.9	33.5	24.7
41A	40.7	46.2	100.0	91.7	65.6	25.9	20.2
41B	46.2	47.9	83.6	100.0	65.8	33.3	21.7
41C	48.3	45.8	74.9	84.6	100.0	40.8	23.5
44	34.5	39.8	19.2	31.1	43.6	100.0	26.5
62	48.4	36.3	43.4	39.4	45.9	34.4	100.0

## Discussion

In this study, we were able to predict, with great accuracy, an eventual recurrence of CDI following cap-FMT at 7 days post-FMT using an unbiased, statistical model incorporating the abundances of members of the families *Lachnospiraceae*, *Ruminococcaceae*, *Bacteroidaceae*, *Porphyromonadaceae*, and *Enterobacteriaceae*. The abundances of *Bacteroides* spp. have been previously suggested to prevent recurrence [13], and the results of our current work support this supposition. However, while we previously suggested that dysbiotic signatures, e.g., sustained, elevated abundances of *Enterobacteriaceae* [18], may be useful in predicting recurrences, our analysis of recurrence events following cap-FMT here did not reflect this. While sustained levels of *Enterobacteriaceae* were noted following some recurrences, others were characterized by near elimination of this family, but incomplete restoration of diversity within the *Firmicutes*, *Bacteroidetes*, or both. This result suggests that the microbial community dynamics surrounding recurrence may be specific to the individual patient [8, 9].

The mechanisms by which FMT resolves rCDI are broadly associated with reinstatement of intestinal microbial diversity, as well as restoration of the functional and beneficial effects of the microbiota on host physiology and gut chemistry [21]. Previous studies have noted significantly reduced alpha diversity that is restored following FMT [17, 22–24], suggesting that competition for nutrients between the reinstated flora and *C. difficile* may play a role in suppressing the infection. Furthermore, a recent longitudinal study found that intestinal microbial diversity was associated with both recurrence of CDI as well as the severity of the disease, with greater diversity associated with decreased severity and reduced likelihood of recurrence [11]. In a mouse model, a single species, *C. scindens*, was inhibitory to *C. difficile* [25], and the suppressive mechanism was found to be associated with secondary bile acid biosynthesis. Similarly, our inferred functional data indicated a positive correlation between time following cFMT and genes associated with secondary bile acid synthesis. Moreover, the restoration of secondary bile acid metabolism was noted in patients treated with FMT [7]. Several studies have also demonstrated the inhibitory effects of some bile acids, including chenodeoxycholic, lithocholic, and ursodeoxycholic acids, on germination of *C. difficile* spores [26–28]. Interestingly, co-administration of bacterial species from *Lachnospiraceae* and *Porphyromonadaceae* families enhanced the protective potency of *C. scindens* against CDI [25]. Thus, the reinstatement of both microbial diversity and specific functional capabilities related to host physiology and microbe-microbe interactions are vital to the efficacy of FMT and restoration of gut health. However, accurate characterization of functions associated with response to FMT will require further experimental characterization.

We employed a regression tree-based machine-learning algorithm in order to assess factors that may be associated with recurrence [29, 30]. Due to the complexity of the microbial dataset, we selected independent variables for this model based on prior statistical analyses to identify highly discriminant taxa, which provided high sensitivity and specificity to detect recurrence at later time points. Thus, this method, using data-specific and statistically derived taxa as independent variables, may allow similar models to be applied to diverse patient populations. While the search for predictive models of recurrence is an active area of research [31], results using our exhaustive CHAID-regression tree model provide much greater accuracy than a random forest-based method used to predict or determine signatures of dysbiosis in microbiome data (maximum classification accuracy of 85.4%) [32]. Furthermore, using unbiased variable selection resulted in high predictive accuracy independent of donor-specific taxa that likely engrafted successfully from some donor lots, such as *Verrucomicrobia*. This suggests that donor engraftment by itself is not solely predictive of the success of FMT, similar to our previous findings using colonoscopic FMT [10].

Full normalization of bacterial community structure following FMT with orally administered, encapsulated, freeze-dried microbiota appears to be significantly delayed relative to that seen with colonoscopic FMT, where donor-like microbiome restoration can be seen as early as 24 h following application [17]. This delay may, in part, be due to variability in location of capsule release of microbiota in different patients given the substantial range in gastric pH and intestinal transit times. Further optimization of microbiota delivery with encapsulated preparations should solve this problem. Currently, our ability to predict failure from a sample obtained at 7 days after FMT, which generally precedes recurrence of CDI symptoms, is already potentially useful clinically. Patients with rCDI syndrome are often discouraged by multiple failures of standard antibiotic therapy and may choose indefinite treatment with vancomycin, despite the expense and risks of furthering antibiotic resistance. A demonstration of incomplete engraftment following cap-FMT suggests a rationale for performing repeat cap-FMTs. Notably, some patients fail even colonoscopic FMT. Unfortunately, we do not have a large systematic collection of stool samples over time following colonoscopic FMT. It is possible that engraftment of some bacteria that support resistance to *C. difficile* is intrinsically difficult due to some yet unknown host-specific factors. Therefore, continued investigations in patients who fail FMT may help identify some of the key bacteria and potential functions that can improve the consistency of next-generation FMT products.

Finally, the SourceTracker analysis done here revealed that complete donor transfer did not occur in patients, similar to previous reports [10, 18], although significantly greater engraftment was observed among responders. We found that the similarity of microbial community structure in patients relative to the donor lot used for FMT reached a maximum around 40–50%. Through the first month of FMT, however, the patient similarity to a broader pool of healthy donors was approximately 20–30% greater. Similarly, within-donor analyses showed, maximally, only 40 to 50% of the microbial community (to as low as 20%) was shared between healthy individuals. This result highlights the intrapersonal variability in the composition of gut microbiota [17, 33] and is suggestive that formulation of the cap-FMT consortium should focus on use of keystone bacterial species, likely those within the families identified, that promote the healthy reorganization of the intestinal microbiota, independent of donor- or patient-specific factors.

## Conclusions

Fecal microbiota transplantation using freeze-dried, encapsulated, microbiota is an effective treatment for rCDI that results in restoration of microbial diversity and reinstatement of healthy microbiota, similar to that observed following colonoscopic and nasoduodenal approaches with frozen or fresh fecal microbiota [16]. While the restoration of bacterial diversity is currently slower than that observed using more traditional approaches [18], shifts in the microbiota reflective of clinical response can be observed within 7 days of cap-FMT. Using a regression tree-based approach, taxa that are predictive of clinical response can be identified and targeted to improve microbial therapeutics. Furthermore, we found that fecal microbiota transfer from donors accounted for approximately half of the patient community among patients who responded to cap-FMT, but that the microbiota then continued to shift to achieve a stable configuration that was more similar to a universal healthy intestinal assemblage. These results demonstrate the efficacy of an unbiased statistical model to determine which taxa are associated with patient response and inform efforts to optimize the cap-FMT preparation.

## Methods

### Preparation of encapsulated microbiota

Encapsulated fecal microbiota was prepared using eight different fecal samples from six donors [numbers 06, 20, 41 (three lots), 42, 44, and 62] who enrolled in the University of Minnesota donor program, as described previously [34]. Any single course of capsules was constituted from only one lot (a single donation) of donor material. Freeze-dried preparations were encapsulated as described previously [15]. Briefly, fecal material was homogenized by blending under N_2_ gas, sieved to remove large particles, amended with 5% trehalose, and freeze-dried. Capsules were stored at − 80 °C until provided to patients. An evolving study protocol was used that was adapted based on clinical experience [15]. The principle factors that were varied, besides donor lot, included (1) total dosage of microorganisms, ranging from 2.1 × 10^11^ to 2.0 × 10^12^ cells, and (2) number of capsules and days over which capsules were taken, ranging from two capsules in a single day to 27 capsules over 3 days of administration (Additional file 1: Table S2).

### Patients and sample collection

Patient inclusion and exclusion criteria, as well as a subset of patient demographics, were described previously [15]. Patients were enrolled if they had at least two prior recurrences of *C. difficile* infection, failed to respond to antibiotic therapies, and were *C.-difficile-*toxin-B-positive by PCR, at least 3 months prior to treatment [15]. This work extends our previous work with the inclusion of a total of 89 patients who received a total of 100 cap-FMTs. The patients were taking oral vancomycin until 2 days prior to cap-FMT treatment. Patients received no colon purgative prior to cap-FMT, as described previously [15]. The FMT capsules were delivered to the patients by a research coordinator. Cap-FMT (2–5 capsules, depending on the preparation lot, as a single treatment dose) was administered on an empty stomach with only clear liquids allowed afterwards for 2 h. The patients remained in close contact with the coordinator and clinical staff throughout follow-up. Clinical failure of cap-FMT was determined as return of diarrheal symptoms and a positive test for *C. difficile* toxin (toxin B PCR) through a 2-month clinical follow-up [15]. Patients who had a recurrence of infection had the option of receiving a follow-up cap-FMT or colonoscopic FMT. The cohort described in this paper includes only the patients who were able to collect the fecal samples for this study. An additional 15 patients treated with cap-FMT did not participate in the stool analysis study because of logistic difficulties or inability to consent. Nevertheless, the clinical outcomes from these patients are included in the Additional file 1.

Patient samples were collected in single-use toilet hats and transferred by the patients to 30 ml polystyrene fecal specimen containers (Globe Scientific, Inc., Paramus, NJ, USA). Samples were stored in the patients’ freezers prior to transport to the laboratory on dry ice, where they were stored at − 20 to − 80 °C prior to DNA extraction.

### DNA extraction and sequencing

DNA was extracted from 250 to 500 mg of thawed donor and patient fecal samples using the DNeasy® PowerSoil® Kit (QIAGEN, Hilden, Germany), according to the manufacturer’s instructions. Fecal material from donor 42 was not available for DNA extraction and sequencing. The V5-V6 hypervariable regions of the 16S rRNA gene were amplified using the BSF784/1064R primer set [35]. Amplification was performed by the University of Minnesota Genomics Center (UMGC, Minneapolis, MN, USA) using an initial amplification with primers including Nextera adapter sequences (Illumina, Inc., San Diego, CA, USA) for 25 cycles. An additional 10 cycles of amplification was performed to add dual index tags to forward and reverse reads [36]. Samples were sized-selected and pooled in equal amounts, as previously described [36], followed by paired-end sequencing at a read length of 300 nucleotides (nt) on the Illumina MiSeq or 250 nt on the HiSeq2500. Negative (sterile water) controls were carried through amplification and sequencing and did not produce amplicons.

### Bioinformatics

Sequence processing and analyses were performed using mothur software ver. 1.35.1 [37], and the batch commands used are available in Additional file 2. Forward and reverse reads were trimmed to 150 nt to eliminate low-quality 3′-regions and paired-end joined using fastq-join software [38]. Joined reads were quality trimmed at a base score of 35 over a window of 50 nt. In addition, samples with ambiguous bases, homopolymers > 8 nt, and > 2 mismatches from primer sequences were removed. High-quality sequences were aligned against the SILVA database ver. 132 [39]. A 2% pre-cluster was used to remove any remaining likely sequence errors [40], and chimeric sequences were identified and removed using UCHIME software ver. 4.2.40 [41]. Operational taxonomic units were assigned at 97% similarity using complete-linkage clustering and taxonomic classification was performed against the version 16 data release from the Ribosomal Database Project [42]. For comparisons among samples, the number of sequences per sample was rarefied to 11,000 sequences by random subsampling [43].

To determine potential structure-function relationships and to examine changes in abundances of traits, functional inferences were made using the PICRUSt (Phylogenetic Investigation of Communities by Reconstruction of Unobserved States) software ver. 1.1.3 [44]. Analyses were done using the Numpy (1.13.3), biom-format (2.1.6), and PyCogent (1.5.3) dependencies. Rarefied sequence data were aligned against the GreenGenes database ver. 13.5 [45] and normalized by copy number. Taxa that contributed to abundances of secondary bile acid genes were determined using the metgenome_contributions.py script. The mean nearest sequence taxon index (NSTI) value among all samples was 0.046 ± 0.022.

SourceTracker ver. 0.9.8 was used to assess engraftment, as the percent of a patient (sink) community that could be attributed to a donor (source) community using a Bayesian inference approach [20]. For all SourceTracker analyses, default parameters were maintained including those for rarefaction (to 1000 reads) and α_1_, α_2_, and β Dirichlet hyperparameters (0.001, 0.01, and 0.01, respectively). To assess how differences in donor pools might affect SourceTracker results, engraftment was calculated by (1) specifying all donor lots as a composite source, with all patient samples as a sink (termed “composite donor”); (2) specifying each donor lot as a unique source, with all patient samples as a sink (termed “specific donor”); and (3) separating samples based on donor lot, where a single donor lot was the source and only samples from patients who received that lot were sinks (termed “individual donor”).

### Statistical analyses

The Shannon index of alpha diversity was calculated by using mothur software. Analysis of similarity (ANOSIM), to assess differences in community composition [46], and ordination by principal coordinate analysis (PCoA) [47] were done by using Bray-Curtis dissimilarity matrices [48]. To determine OTUs significantly associated with PCoA axis position, the corr.axes function in mothur was used. Linear discriminant analysis of effect size (LEfSe) [49] was used to evaluate OTUs indicative of clinical outcome at specific time points.

A regression tree approach was used to determine whether it was possible to build a predictive model for the detection of recurrence. In this approach, the exhaustive chi-squared automatic interaction detection (CHAID) procedure [29] was utilized, with the Pearson measure set at a maximum tree depth of five levels. Ten samples from the training data were withheld for internal model validation. The CHAID-regression tree model was built and tested using XLSTAT software ver. 17.06 (Addinsoft, Belmont, MA), with all other default settings maintained. Input data consisted of the relative abundances (as percent) of the families among all 7-day-post-FMT time points. Following training, the model was tested against all patient samples not used in the training set) from days 8 to 20. For follow-up testing and potential tuning, all tree-building parameters were maintained. The tree structure obtained to predict recurrence is shown in Additional file 1: Table S5.

Chi-squared tests, analysis of variance (ANOVA), followed by Tukey’s *post hoc* test, and Spearman rank correlations were also performed using XLSTAT. All statistics were evaluated at *α* = 0.05 with Bonferroni correction for multiple comparisons, where applicable.

## Additional files

Additional file 1: Supplemental figures and tables. (DOCX 292 kb)

Additional file 2: Batch file for sequencing processing. (TXT 12 kb)

## Abbreviations

ANOSIM: Analysis of similarity
cap-FMT: Encapsulated fecal microbiota transplantation
CDI: *Clostridium difficile* infection
FMT: Fecal microbiota transplantation
LEfSe: Linear discriminant analysis of effect size
OTU: Operational taxonomic unit
PCoA: Principal coordinate analysis
rCDI: Recurrent *Clostridium difficile* infection
