# Supplementary material for: Predicting recurrence of Clostridium difficile infection following encapsulated fecal microbiota transplantation
Source: Microbiome. 2018 Sep 18;6:166. doi: 10.1186/s40168-018-0549-6 (PMC6145197; doi:10.1186/s40168-018-0549-6)
Supplement: Supplementary file 1 — Supplemental figures and tables. (DOCX 292 kb) [file 40168_2018_549_MOESM1_ESM.docx]

**Supplemental Figures and Tables**

# Predicting *Clostridium difficile* Recurrence following Encapsulated Fecal Microbiota Transplantation

Christopher Staley^1,2^, Thomas Kaiser^1,2^, Byron P. Vaughn^3^, Carolyn T. Graiziger^3^, Matthew J. Hamilton^2^, Tauseef ur Rehman^3^, Kevin Song^3^, Alexander Khoruts^2,3,*^, and Michael J. Sadowsky^2,4,5,*,#^

^1^Department of Surgery, University of Minnesota, Minneapolis, Minnesota, USA

^2^BioTechnology Institute, University of Minnesota, St. Paul, Minnesota

^3^Division of Gastroenterology, Department of Medicine, University of Minnesota, Minneapolis, Minnesota, USA

^4^Department of Soil, Water and Climate, University of Minnesota, St. Paul, Minnesota, USA

^5^Department of Plant and Microbial Biology, University of Minnesota, St. Paul, Minnesota, USA

*Shares senior authorship.

Running Title: Prediction of recurrence following cap-FMT

^#^Corresponding Author: Michael J. Sadowsky, BioTechnology Institute, University of Minnesota, 140 Gortner Lab, 1479 Gortner Ave, Saint Paul, MN 55108; Phone: (612)-624-2706, Email: sadowsky@umn.edu

**
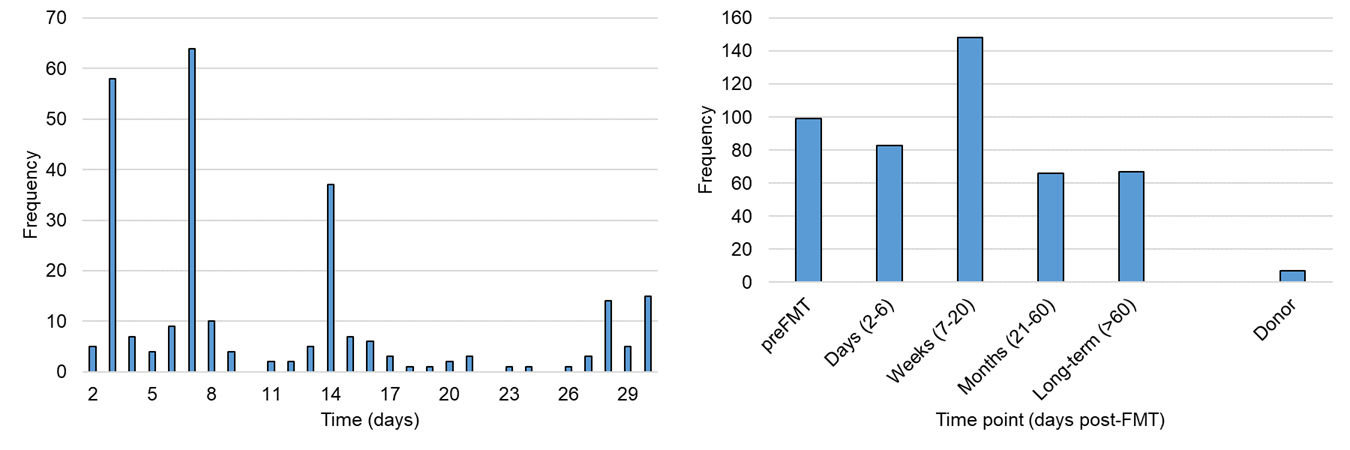
**

**Figure S1** – Histograms of sample collection frequency among patients treated with cap-FMT. A) Frequency of sample collection within the first 30 days post-FMT. B) Frequency of sample collection by temporal category, where numbers in parentheses indicate the numbers of days post-FMT captured in each category.


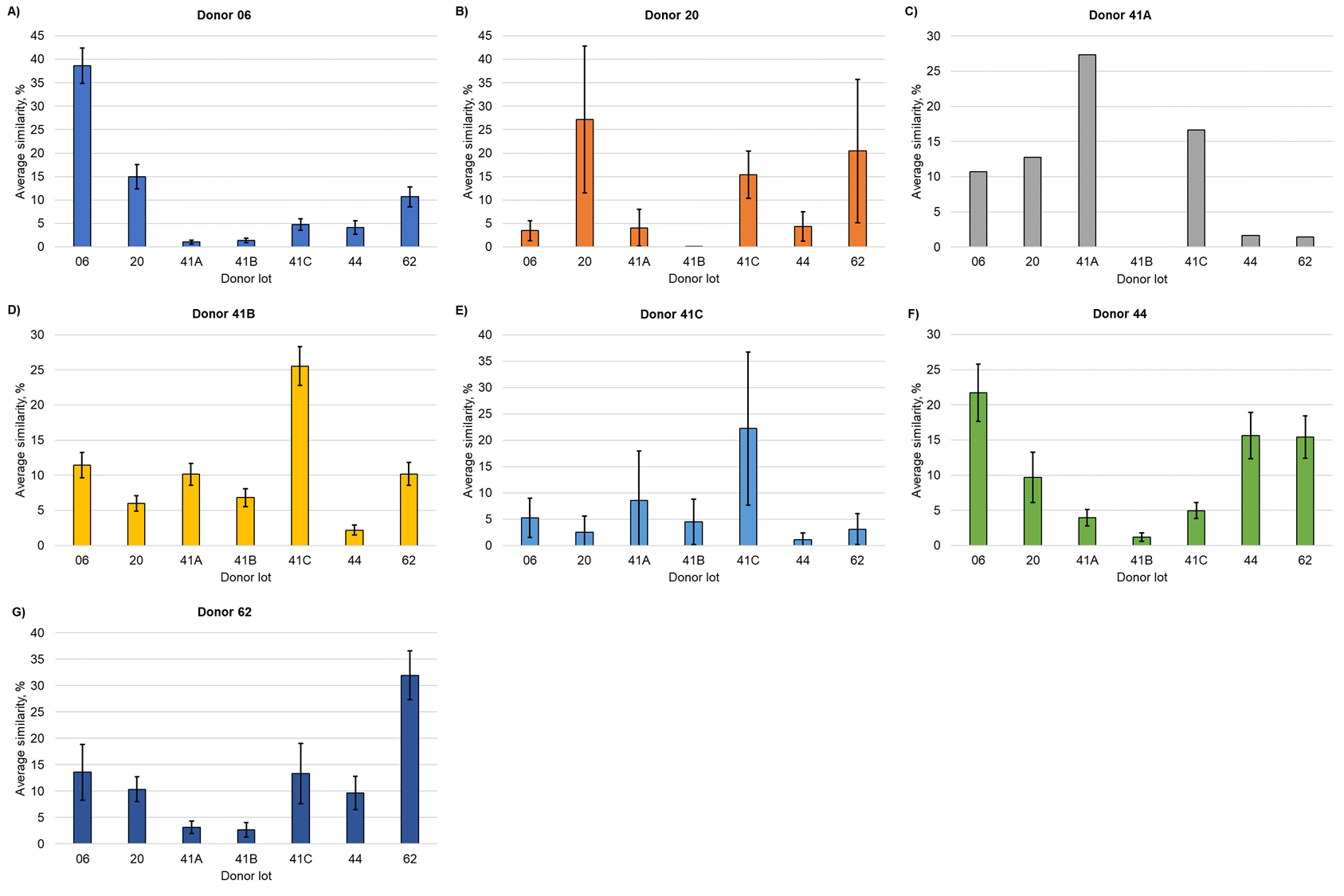


**Figure S2 –** Similarity of bacterial communities to specific donor lots among samples from responders collected at the ‘weeks’ time point. Samples are separated by the donor lot received: A) donor 06, B) donor 20, C) donor 41A, D) donor 41B, E) donor 41C, F) donor 44, and G) donor 62. Error bars reflect SEM.

**Table S1** – Demographics and clinical characteristics of the patient cohort included in the stool analysis study.^1^

| Age (years ± SD) | 63 ± 18 |
| --- | --- |
| Sex (% female) | 85 |
| History of hospitalization for severe or severe-complicated CDI (%) | 33 |
| Median number of months between the initial CDI and cap-FMT treatment (range) | 10 (3 – 47) |

^1^Fifteen additional patients not included in the stool analysis study were treated with cap-FMT. Common reasons for exclusion from the study included (1) inability to consent due to dementia or a psychiatric condition, (2) logistic considerations such as severe physical disability and lack of necessary caretaker assistance, and (3) remoteness from the research center. 13/15 patients (86.7%) had successful outcomes of cap-FMT (no recurrence of CDI within 2 months of follow-up).

**Table S2 –** Patient treatment information related to clinical outcome. A) Donor lot, B) capsule dosage (total cells), and C) capsule delivery method (capsules/day).

| **A)** | **Donor**  **lot** | **Response** | **Recurrence** | **B)** | **Total**  **dosage^*^** | **Response** | **Recurrence** | **C)** | | **Delivery**  **method**^†^ | **Response** | **Recurrence** |
| --- | --- | --- | --- | --- | --- | --- | --- | --- | --- | --- | --- | --- |
|  | 06 | 18 | 5 |  | 2.10 × 10^11^ | 24 | 7 |  | | 2/1 | 17 | 6 |
|  | 20 | 3 | 0 |  | 2.50 × 10^11^ | 14 | 1 |  | | 4/1 | 30 | 5 |
|  | 41A | 1 | 0 |  | 4.70 × 10^11^ | 6 | 2 |  | | 5/1 | 7 | 2 |
|  | 41B | 24 | 7 |  | 5.00 × 10^11^ | 18 | 5 |  | | 10/1 | 7 | 3 |
|  | 41C | 3 | 2 |  | 1.25 × 10^12^ | 5 | 3 |  | | 12/1 | 6 | 1 |
|  | 42 | 6 | 2 |  | 2.00 × 10^12^ | 4 | 0 |  | | 14/1 | 3 | 2 |
|  | 44 | 16 | 2 |  |  |  |  |  | | 20/1 | 1 | 1 |
|  | 62 | 7 | 3 |  |  |  |  |  | | 27/2-3 | 4 | 0 |
|  | *χ*^2^ = 3.801, *P* = 0.802 | | |  | *χ*^2^ = 4.455, *P* = 0.486 | | | |  | *χ*^2^ = 5.152, *P* = 0.641 | | |

^*^Ten capsule FMTs were performed at an undetermined concentration.

^†^Number of capsules/number of days taken

**Table S3 –** Significant correlations (*P* < 0.05) observed between time-post-FMT (days) and abundances of inferred metabolic functional genes, annotated against the KEGG orthology (KO), among patients who responded to cFMT.

| **KO Tier 2** | **KO Tier 3** | **Spearman’s *ρ* (*P*-value)** |
| --- | --- | --- |
| Amino acid metabolism | Glycine, serine and threonine metabolism | -0.121 (0.043) |
|  | Lysine degradation | -0.347 (< 0.0001) |
|  | Phenylalanine metabolism | -0.220 (< 0.0001) |
|  | Tryptophan metabolism | -0.325 (< 0.0001) |
|  | Tyrosine metabolism | -0.196 (0.001) |
|  | Valine, leucine and isoleucine degradation | -0.367 (< 0.0001) |
| Biosynthesis of other secondary metabolites | Clavulanic acid biosynthesis | -0.144 (0.016) |
|  | Flavone and flavonol biosynthesis | 0.173 (0.004) |
|  | Isoflavonoid biosynthesis | -0.145 (0.015) |
|  | Isoquinoline alkaloid biosynthesis | -0.217 (< 0.0001) |
|  | Penicillin and cephalosporin biosynthesis | -0.124 (0.039) |
|  | Stilbenoid, diarylheptanoid and gingerol biosynthesis | -0.191 (0.001) |
|  | Tropane, piperidine and pyridine alkaloid biosynthesis | -0.162 (0.007) |
| Carbohydrate metabolism | Ascorbate and aldarate metabolism | -0.309 (< 0.0001) |
|  | Butanoate metabolism | -0.238 (< 0.0001) |
|  | Citrate cycle (TCA cycle) | -0.145 (0.015) |
|  | Fructose and mannose metabolism | -0.126 (0.035) |
|  | Glycolysis / Gluconeogenesis | -0.134 (0.025) |
|  | Glyoxylate and dicarboxylate metabolism | -0.158 (0.008) |
|  | Inositol phosphate metabolism | -0.302 (< 0.0001) |
|  | Propanoate metabolism | -0.183 (0.002) |
| Energy metabolism | Nitrogen metabolism | -0.124 (0.038) |
|  | Sulfur metabolism | -0.158 (0.008) |
| Enzyme families | Cytochrome P450 | -0.170 (0.004) |
|  | Protein kinases | -0.161 (0.007) |
| Glycan biosynthesis and metabolism | Glycosaminoglycan degradation | 0.132 (0.027) |
|  | Glycosphingolipid biosynthesis - ganglio series | 0.147 (0.014) |
|  | Glycosphingolipid biosynthesis - globo series | 0.139 (0.020) |
|  | Glycosyltransferases | -0.224 (< 0.0001) |
|  | Lipopolysaccharide biosynthesis | -0.175 (0.003) |
|  | Lipopolysaccharide biosynthesis proteins | -0.211(< 0.0001) |
|  | N-Glycan biosynthesis | 0.125 (0.037) |
|  | Other glycan degradation | 0.191 (0.001) |
| Lipid metabolism | alpha-Linolenic acid metabolism | -0.282 (< 0.0001) |
|  | Arachidonic acid metabolism | -0.320 (< 0.0001) |
|  | Biosynthesis of unsaturated fatty acids | -0.247 (< 0.0001) |
|  | Fatty acid metabolism | -0.254 (< 0.0001) |
|  | Primary bile acid biosynthesis | 0.215 (< 0.0001) |
|  | Secondary bile acid biosynthesis | 0.217 (< 0.0001) |
|  | Sphingolipid metabolism | 0.131 (0.029) |
|  | Synthesis and degradation of ketone bodies | -0.309 (< 0.0001) |
| Metabolism of cofactors and vitamins | Lipoic acid metabolism | -0.163 (0.006) |
|  | Retinol metabolism | -0.270 (< 0.0001) |
|  | Riboflavin metabolism | -0.192 (0.001) |
|  | Ubiquinone and other terpenoid-quinone biosynthesis | -0.216 (< 0.0001) |
| Metabolism of other amino acids | beta-Alanine metabolism | -0.182 (0.002) |
|  | D-Alanine metabolism | -0.139 (0.020) |
|  | D-Arginine and D-ornithine metabolism | -0.261 (< 0.0001) |
|  | Glutathione metabolism | -0.361 (< 0.0001) |
|  | Phosphonate and phosphinate metabolism | -0.137 (0.022) |
|  | Selenocompound metabolism | -0.126 (0.035) |
|  | Taurine and hypotaurine metabolism | -0.154 (0.01) |
| Metabolism of terpenoids and polyketides | Biosynthesis of 12-, 14- and 16-membered macrolides | -0.145 (0.015) |
|  | Biosynthesis of ansamycins | -0.124 (0.038) |
|  | Biosynthesis of siderophore group nonribosomal peptides | -0.415 (< 0.0001) |
|  | Biosynthesis of type II polyketide backbone | -0.144 (0.016) |
|  | Biosynthesis of vancomycin group antibiotics | 0.159 (0.008) |
|  | Carotenoid biosynthesis | -0.217 (< 0.0001) |
|  | Geraniol degradation | -0.252 (< 0.0001) |
|  | Limonene and pinene degradation | -0.292 (< 0.0001) |
|  | Polyketide sugar unit biosynthesis | 0.130 (0.030) |
|  | Sesquiterpenoid biosynthesis | -0.145 (0.016) |
|  | Zeatin biosynthesis | 0.127 (0.034) |
| Xenobiotics biodegradation and metabolism | Aminobenzoate degradation | -0.281 (< 0.0001) |
|  | Benzoate degradation | -0.286 (< 0.0001) |
|  | Caprolactam degradation | -0.285 (< 0.0001) |
|  | Chlorocyclohexane and chlorobenzene degradation | -0.240 (< 0.0001) |
|  | Dioxin degradation | -0.210 (< 0.0001) |
|  | Drug metabolism - cytochrome P450 | -0.286 (< 0.0001) |
|  | Ethylbenzene degradation | -0.170 (0.004) |
|  | Fluorobenzoate degradation | -0.273 (< 0.0001) |
|  | Metabolism of xenobiotics by cytochrome P450 | -0.303 (< 0.0001) |
|  | Naphthalene degradation | -0.181 (0.002) |
|  | Styrene degradation | -0.263 (< 0.0001) |
|  | Toluene degradation | -0.124 (0.038) |
|  | Xylene degradation | -0.143 (0.017) |

**Table S4 –** Family-level classification of OTUs found to be transferred from individual donor lots to patients receiving that lot by SourceTracker. The ‘weeks’ time point is shown (mean ± SEM).

| **Donor^*^** | **Clinical outcome** | ***n*** | ***Lachnospiraceae*** | ***Ruminococcaceae*** | ***Bacteroidaceae*** | ***Porphyromonadaceae*** | ***Verrucomicrobiaceae*** | ***Enterobacteriaceae*** |
| --- | --- | --- | --- | --- | --- | --- | --- | --- |
| 06 | Response | 30 | 23.5 ± 3.5 | 5.8 ± 1.1 | 7.5 ± 1.9 | 6.6 ± 1.7 | 0.0 ± 0.0 | 0.1 ± 0.0 |
|  | Recurrence | 6 | 27.4 ± 10.9 | 4.2 ± 2.7 | 6.2 ± 4.9 | 10.7 ± 8.2 | 0.0 ± 0.0 | 0.3 ± 0.1 |
| 20^†^ | Response | 3 | 28.5 ± 20.0 | 5.1 ± 4.3 | 4.2 ± 2.8 | 2.3 ± 2.9 | 0.0 ± 0.0 | 3.6 ± 2.7 |
| 41A^†^ | Response | 1 | 12.9 | 2.6 | 25.6 | 0.1 | 0 | 0 |
| 41B | Response | 38 | 13.5 ± 1.9 | 5.0 ± 0.8 | 7.5 ± 1.8 | 5.1 ± 1.2 | 0.0 ± 0.0 | 0.0 ± 0.0 |
|  | Recurrence | 13 | 7.8 ± 3.1 | 1.8 ± 0.8 | 1.2 ± 1.1 | 8.0 ± 3.3 | 0.0 ± 0.0 | 0.0 ± 0.0 |
| 41C | Response | 3 | 11.5 ± 7.7 | 5.6 ± 6.3 | 1.0 ± 1.2 | 0.0 ± 0.0 | 0.0 ± 0.0 | 0.0 ± 0.0 |
|  | Recurrence | 2 | 10.6 ± 12.6 | 3.1 ± 4.0 | 15.3 ± 9.6 | 0.0 ± 0.0 | 0.0 ± 0.0 | 0.0 ± 0.0 |
| 44 | Response | 21 | 17.5 ± 3.4 | 3.7 ± 1.0 | 6.4 ± 2.5 | 0.6 ± 0.6 | 7.6 ± 2.2 | 0.0 ± 0.0 |
|  | Recurrence | 3 | 21.0 ± 4.8 | 1.2 ± 0.7 | 1.6 ± 1.5 | 0.2 ± 0.2 | 0.2 ± 0.2 | 0.0 ± 0.0 |
| 62 | Response | 8 | 12.4 ± 1.7 | 8.6 ± 2.4 | 6.3 ± 2.9 | 1.6 ± 0.8 | 14.1 ± 4.0 | 0.0 ± 0.0 |
|  | Recurrence | 1 | 30.9 | 13.4 | 0.1 | 0.0 | 0.1 | 0 |

^*^Fecal material from donor 42 was not available for sequencing.

^†^No patients receiving this donor lot experienced a recurrence of infection

**Table S5** – Structure of the CHAID-regression tree model built using the families *Lachnospiraceae*, *Ruminococcaceae*, *Bacteroidaceae*, *Porphyromonadaceae*, and *Enterobacteriaceae*. The % purity and event reflect the percent of samples represented by the node that were classified to the designated event, *i.e.*, for the 13 samples that are represented by node 2, which have abundances of *Ruminococcaceae* between 0.0 – 3.1%, 53.8% were associated with recurrence.

| **Node** | ***p*-value** | **Objects** | **Parent node** | **Split variable** | **Values (% low, high)** | **Purity** | **Event** |
| --- | --- | --- | --- | --- | --- | --- | --- |
| 1 | 0.366 | 54 |  |  |  | 81.5% |  |
| 2 | 0.615 | 13 | 1 | *Ruminococcaceae* | 0.0, 3.1 | 53.8% | Recurrence |
| 3 | 0.543 | 7 | 1 | *Ruminococcaceae* | 3.1, 6.1 | 85.7% | Response |
| 4 | 0.000 | 9 | 1 | *Ruminococcaceae* | 6.1, 9.5 | 100.0% | Response |
| 5 | 1.000 | 9 | 1 | *Ruminococcaceae* | 9.5, 13.1 | 88.9% | Response |
| 6 | 0.000 | 6 | 1 | *Ruminococcaceae* | 13.1, 17.5 | 100.0% | Response |
| 7 | 1.000 | 2 | 1 | *Ruminococcaceae* | 17.5, 20.8 | 50.0% | Recurrence |
| 8 | 0.000 | 8 | 1 | *Ruminococcaceae* | 20.8, 33.5 | 100.0% | Response |
| 9 | 0.707 | 6 | 2 | *Lachnospiraceae* | 0.0, 16.4 | 50.0% | Recurrence |
| 10 | 0.000 | 3 | 2 | *Lachnospiraceae* | 16.4, 48.4 | 100.0% | Response |
| 11 | 0.000 | 4 | 2 | *Lachnospiraceae* | 48.4, 85.4 | 100.0% | Recurrence |
| 12 | 0.577 | 4 | 9 | *Enterobacteriaceae* | 0.4, 25.2 | 75.0% | Recurrence |
| 13 | 0.000 | 2 | 9 | *Enterobacteriaceae* | 25.2, 94.0 | 100.0% | Response |
| 14 | 0.000 | 2 | 12 | *Lachnospiraceae* | 0.0, 8.7 | 100.0% | Recurrence |
| 15 | 1.000 | 2 | 12 | *Lachnospiraceae* | 8.7, 16.4 | 50.0% | Recurrence |
| 16 | 0.000 | 1 | 15 | *Porphyromonadaceae* | 0.0, 22.6 | 100.0% | Response |
| 17 | 0.000 | 1 | 15 | *Porphyromonadaceae* | 22.6, 45.1 | 100.0% | Recurrence |
| 18 | 0.000 | 1 | 7 | *Porphyromonadaceae* | 0.0, 14.4 | 100.0% | Response |
| 19 | 0.000 | 1 | 7 | *Porphyromonadaceae* | 14.4, 28.8 | 100.0% | Recurrence |
| 20 | 0.000 | 1 | 3 | *Lachnospiraceae* | 12.5, 14.6 | 100.0% | Response |
| 21 | 1.000 | 2 | 3 | *Lachnospiraceae* | 14.6, 29.1 | 50.0% | Recurrence |
| 22 | 0.000 | 4 | 3 | *Lachnospiraceae* | 29.1, 80.6 | 100.0% | Response |
| 23 | 0.000 | 1 | 21 | *Bacteroidaceae* | 4.8, 17.4 | 100.0% | Recurrence |
| 24 | 0.000 | 1 | 21 | *Bacteroidaceae* | 17.4, 30.0 | 100.0% | Response |
| 25 | 0.000 | 1 | 5 | *Lachnospiraceae* | 10.9, 17.6 | 100.0% | Recurrence |
| 26 | 0.000 | 8 | 5 | *Lachnospiraceae* | 17.6, 78.9 | 100.0% | Response |
